# Implementation of Photosynaptic and Electrical Memory Functions in Organic Nano‐Floating‐Gate Transistors via a Perovskite‐Nanocrystal‐Based Nanocomposite Tunneling Layer

**DOI:** 10.1002/smsc.202300068

**Published:** 2023-07-18

**Authors:** Byung Joon Moon, Young-Seok Song, Dabin Son, Hee Yun Yang, Sukang Bae, Seoung-Ki Lee, Sang Hyun Lee, Tae-Wook Kim

**Affiliations:** ^1^ Functional Composite Materials Research Center Institute of Advanced Composite Materials Korea Institute of Science and Technology 92 Chudong-ro, Bongdong-eup Wanju-gun Jeollabuk-do 55324 Republic of Korea; ^2^ Department of JBNU-KIST Industry-Academia Convergence Research Jeonbuk National University 567 Baekje-daero, Deokjin-gu Jeonju 54896 Republic of Korea; ^3^ Department of Flexible and Printable Electronics LANL-JBNU Engineering Institute-Korea Jeonbuk National University 567 Baekje-daero Deokjin-gu Jeonju 54896 Republic of Korea; ^4^ School of Chemical Engineering Chonnam National University 77 Yongbong-ro Buk-gu Gwangju 61186 Republic of Korea; ^5^ School of Materials Science and Engineering Pusan National University 2, Busandaehak-ro-63-beon-gil, Geumjeong-gu Busan 46241 Republic of Korea

**Keywords:** nanocomposites, memory, organic nano-floating gate transistors, photosynaptic, provskite nanocrystals

## Abstract

An organic nano‐floating‐gate transistor (ONFGT) with both photosynaptic and electrical memory functions is developed using a perovskite (CsPbBr_3_) NC‐insulating polymer (polystyrene; PS) nanocomposite and CsPbBr_3_ NCs as the tunneling and floating gate layers, respectively. The introduction of the CsPbBr_3_ NCs–PS nanocomposite layer improves the photoresponsivity of the ONFGT under ultraviolet–visible irradiation, resulting in an increase in both the photocurrent and the light‐to‐dark current ratio by 10^−8^ A and 10^4^ orders of magnitude, respectively. It also exhibits high responsivity (0.804 A W^−1^) and external quantum efficiency (249.3%) under 400 nm irradiation. Furthermore, the photosynaptic characteristics of the ONFGT under visible‐light irradiation are investigated. To mimic biological nervous systems, the photocurrent of the device is dynamically modulated by varying the light intensity and duration. Notably, an increase in synaptic weight is observed under repeated photonic stimulations, as shown by changes in synaptic weight with each light pulse. Also, the ONFGT exhibits excellent nonvolatile memory characteristics in the dark, displaying a hysteresis window value of 2.9 V for a gate double sweep under ±5.0 V. Consequently, the perovskite NCs–insulating polymer nanocomposite tunneling layer is crucial for enabling photoresponsivity and memory characteristics in nano‐floating‐gate transistors, making them suitable for multifunctional electronic devices.

## Graphical abstract

In this study, a dual‐function organic nano‐floating‐gate transistor (ONFGT) is fabricated by introducing a CsPbBr_3_ nanocrystals (NCs)–polystyrene nanocomposite as the tunneling layer and utilizing CsPbBr_3_ NCs as the floating‐gate layer. This ONFGT demonstrates remarkable properties, including dynamic modulation of the photocurrent through adjustments in light intensity and duration, as well as exceptional nonvolatile memory characteristics.

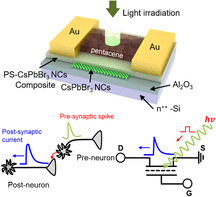

## 1 Introduction

In recent times, organic electronic devices have emerged as a promising technology for soft electronics.^[^ ^1^ ^]^ Significant efforts have been made to develop efficient organic electronic components with high performance and diverse functions by introducing various functional materials into the conventional device platform.^[^ ^2^, ^3^, ^4^ ^]^ The existing electronic device platforms, such as diodes, capacitors, resistors, and transistors, have been utilized as the main components of electronic circuits.^[^ ^5^, ^6^, ^7^ ^]^ Of all the device platforms, transistors have been widely used to implement complementary circuits, including logic, memory, and sensory systems.^[^ ^8^, ^9^ ^]^ To achieve target‐oriented electronic properties of transistors, numerous studies have been conducted to improve functions or performance (such as mobility, threshold voltage, on/off ratio, operational voltage, hysteresis window, and photoresponsivity) by adding or customizing specific layers while maintaining the device structure.^[^ ^10^, ^11^, ^12^, ^13^ ^]^ It is well known that the performance of a transistor is strongly related to the quality of both the semiconductor and gate dielectrics, as well as the interface between them. Achieving high‐performance transistors with improved quality and reliability requires a combination of careful process control, material selection, and device design.^[^ ^14^, ^15^, ^16^ ^]^

To enable the diverse use of transistors with additional functions, such as photoresponsivity or memory characteristics, zero‐dimensional nanomaterials (e.g., metallic or semiconducting nanocrystals) have been widely used as nanofillers to enhance optoelectronic properties such as charge trapping or light absorption. The incorporation of nanofillers into conventional transistors can be achieved by either mixing them with semiconductors or insulating layers or by using them as independent layers in between.^[^ ^17^, ^18^, ^19^ ^]^ Semiconductor nanocrystals (NCs) differ from metal nanocrystals in their ability to both generate charge when exposed to light and store charge, making them a versatile material for use in transistors that can perform a range of functions.

Among various semiconductor nanocrystals, all‐inorganic perovskite nanocrystals, specifically CsPbX_3_ NCs (where X is a halide), have received great attention due to their outstanding optoelectrical characteristics such as long diffusion length,^[^ ^20^ ^]^ broad absorption window,^[^ ^21^, ^22^ ^]^ high absorption coefficient,^[^ ^23^ ^]^ high photoluminescence (PL) quantum yield,^[^ ^24^ ^]^ tunable bandgap,^[^ ^25^ ^]^ high carrier mobility (≈4500 cm^2^ V^−1^ s^−1^),^[^ ^26^ ^]^ and low exciton binding energies.^[^ ^20^, ^27^ ^]^ It is widely recognized that optical characteristics of CsPbX_3_ NCs are primarily attributed to their electronic structure, which can be readily modified by adjusting the halide composition. To be specific, the conduction band is derived from the antibonding orbitals resulting from the hybridization of Pb 6p orbitals and the outermost p orbitals of each halide (e.g., 3p (Cl), 4p (Br), and 5p (I)), while the valence band is formed by the antibonding states of the hybridization of the Pb 6*s* and the halide p‐orbitals. This phenomenon, in contrast to conventional semiconductor NCs, allows for a larger shift in the energy level of the valence band edge upon modification of the halide component, resulting in significant changes in their absorption/emission characteristics. In addition, the exciton‐binding energies (which are important parameters for an opto‐electronic material) of CsPbX_3_ NCs (i.e., CsPbBr_3_ (40 meV) and CsPbCl_3_ (75 meV)) were estimated to be higher than the thermal ionization energy at room temperature (about 26 meV),^[^ ^27^ ^]^ indicating that the photogenerated excitons are stable above room temperature. On top of that, their comparatively low exciton binding energy values compared with other types of nanomaterials make it feasible to efficiently dissociate photogenerated excitons into free charge carriers driven by the internal electric field in the heterojunction. These low values are responsible for the enhanced optoelectronic performance of the devices. There have been many studies to implement functional optoelectronic devices using perovskite NCs due to the electronic and optical uniqueness of perovskite NCs.^[^ ^28^, ^29^, ^30^ ^]^

A conventional nano‐floating‐gate transistor is composed of the tunneling/nano‐floating‐gate/blocking layer in between the semiconductor and gate electrode.^[^ ^8^ ^]^ Similarly, the perovskite NCs layer was introduced as a floating gate in between insulating polymers, which allows implementing perovskite NCs in a nano‐floating‐gate transistor to provide charge trapping sites.^[^ ^31^, ^32^, ^33^, ^34^ ^]^ This system enables excellent charge storage characteristics and provides huge hysteresis windows in the nano‐floating‐gate transistor by separating the floating gate in between the semiconducting layer and gate electrode. Although perovskite NCs had excellent optical properties described earlier, this conventional sandwich structure does not allow any proper transfer of the photogenerated charge carriers toward a semiconducting layer right after absorption of photon energy. Previous studies primarily focused on implementing photoelectric memory or photosynaptic characteristics by co‐modulating electric and photonic stimuli, namely external electrical and optical signals.^[^ ^31^, ^32^, ^33^, ^34^, ^35^, ^36^ ^]^ However, these studies did not demonstrate independent operations in response to electric or photonic signals. For instance, the device's functionality as electrical memory and photosynaptic function was not demonstrated separately under dark and light illumination, respectively. It is widely recognized that perovskite NCs situated in close vicinity to the semiconductor layer are likely to undergo bimolecular recombination after the charge separation of excitons that are formed by the above‐gap light. This can lead to a decrease in the local trapped electron density in the NCs and ultimately cause degradation of the optoelectrical performance of the transistor.^[^ ^37^, ^38^ ^]^ The nonradiative energy transfer process known as Förster resonance energy transfer (FRET) that occurs through long‐range dipole–dipole interactions between two materials can take place from the perovskite NCs to semiconductor layers within a limited distance range,^[^ ^39^ ^]^ which has a negative effect on nonvolatile memory behavior as it decreases the number of photoinduced carriers generated within the perovskite NCs. Therefore, to enhance the charge transfer and storage characteristics of the perovskite NC nano‐floating‐gate transistor under both light irradiation and gate bias conditions, it is necessary to modify the device architecture by replacing the single component tunneling layer with a functional nanocomposite that enables independent dual‐functionality (including photoresponsivity and nonvolatile memory characteristics) in perovskite NC‐based organic nano‐floating‐gate transistors (ONFGTs).

In this study, we fabricated a dual‐function organic nano‐floating‐gate transistor by introducing a perovskite (CsPbBr_3_) NCs–insulating polymer (polystyrene; PS) nanocomposite and CsPbBr_3_ NCs as the tunneling layer and floating gate layer, respectively. By adding the nanocomposite as a tunneling layer, our perovskite nano‐floating‐gate transistor exhibited photoresponsivity resulting in a significant increase in source to drain current under UV–visible irradiation, implying that the photogenerated charge carriers are transferred from CsPbBr_3_ NCs of the nanocomposite to the organic semiconductor layer. Our device responded to 550 nm visible light, indicating that the photogenerated charge carriers originated from the CsPbBr_3_ NCs in the nanocomposite layer. Based on the photoresponsivity of the device, we examined photosynaptic characteristics against incident visible light. Dynamic modulation of the photocurrent of the device was implemented to mimic biological nervous system by varying the intensity and duration time of the light. By plotting the changes in the synaptic weight for each visible‐light pulse, we clearly observed the increase in synaptic weight (post‐synaptic current, PSC) under repeated photonic stimulation. In addition, the perovskite nano‐floating‐gate transistor exhibited excellent nonvolatile memory characteristics displaying a 2.9 V hysteresis window value (Δ*V*_th_) at the gate bias double sweep under ±5.0 V. Drain current values were well maintained after programing and erasing, exhibiting more than two orders of magnitude for 20 000 s. Based on the above results, we found that the perovskite NCs–insulating polymer nanocomposite tunneling layer is considered a key component for achieving dual functions (photoresponsivity and memory characteristics) in a conventional nano‐floating‐gate transistor.

## 2 Results and Discussion

**Figure 1:**
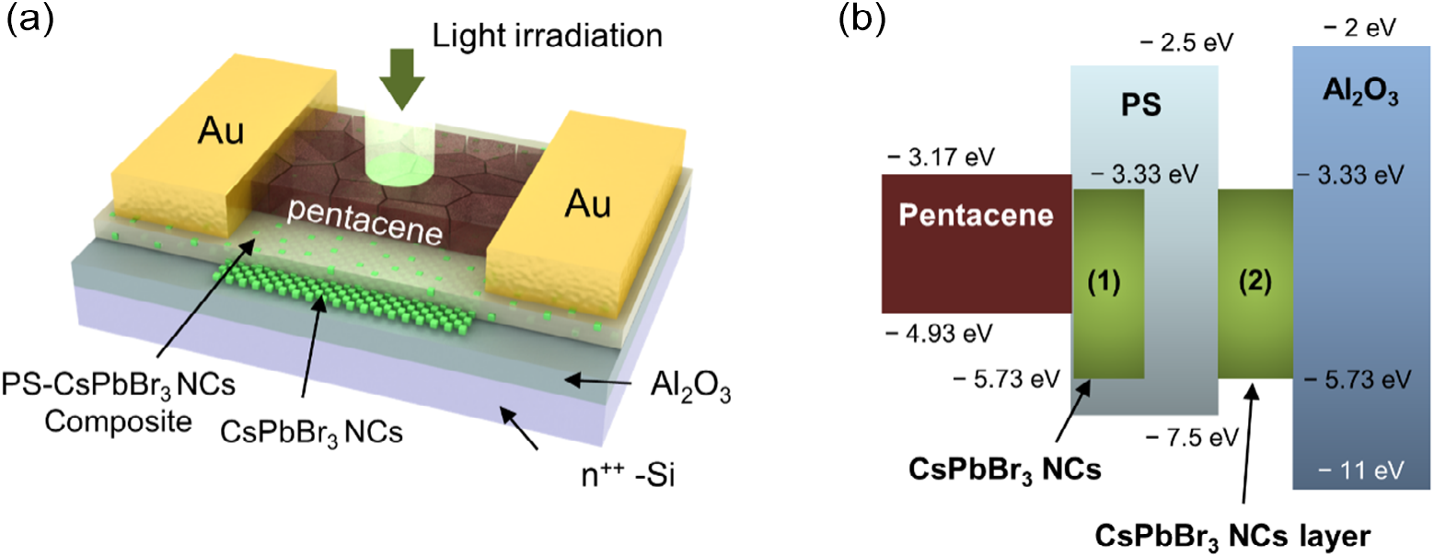
a) Schematic of device structure. b) Energy diagram of the ONFGT with CsPbBr_3_ NCs–PS nanocomposite and CsPbBr_3_ NCs as the tunneling and floating gate layers, respectively.

We used a conventional bottom gate‐top contact thin‐film transistor structure to implement dual‐function ONFGTs, as shown in **Figure** **1**a. The ONFGTs had a similar structure to a typical organic nano‐floating‐gate transistor. However, it also has multifunctionality due to the CsPbBr_3_ NCs–PS nanocomposite and CsPbBr_3_ NC as the tunneling layer and nano‐floating‐gate, respectively. The ONFGTs were fabricated on a heavily doped n‐type Si substrate, which was used as both the substrate and the bottom gate electrode. CsPbBr_3_ NCs served as a nano‐floating‐gate (charge trapping medium) by placing it between the ALD grown Al_2_O_3_ (23 nm) blocking layer and CsPbBr_3_‐PS nanocomposite tunneling layer. Then, pentacene, an organic semiconducting layer, was carefully thermally deposited on the CsPbBr_3_‐PS composite layer. Unlike the conventional configuration of organic‐based nano‐floating‐gate transistors, we employed a CsPbBr_3_‐PS nanocomposite as a tunneling layer in between the organic semiconductor (pentacene) and charge trapping layer (CsPbBr_3_ NCs), as shown in Figure 1a. The energy band diagram of the ONFGTs based on our device structure is shown in Figure 1b. To identify the electronic band structure of the ONFGTs, UV photoelectron spectroscopy (UPS) analysis was carried out on both the pentacene and CsPbBr_3_ NCs thin films, which are carefully prepared on the thermally deposited reference Au thin film (the measured work function *ϕ*_Au_ = 4.89 eV). The measured HOMO level and LUMO levels of thermally deposited pentacene thin film were located at −4.93 and −3.17 eV from the vacuum level, respectively, indicating that the calculated bandgap of the pentacene was 1.76 eV, which is consistent with previous literature.^[^ ^36^ ^]^ On the other hand, the HOMO level and LUMO level of CsPbBr_3_ NCs were measured at −5.73 and −3.33 eV from the vacuum level, respectively, with a bandgap of 2.4 eV. The experimentally measured bandgap of the CsPbBr_3_ NCs could deviate from the theoretically calculated value of the bulk perovskite (*E*_g_ = 2.29 eV) due to the quantum confinement effect, the size, crystal structure, and purification.^[^ ^40^, ^41^ ^]^ The measured energy levels of pentacene and CsPbBr_3_ NCs by UPS were once again verified by optical property measurements described earlier.

**Figure 2:**
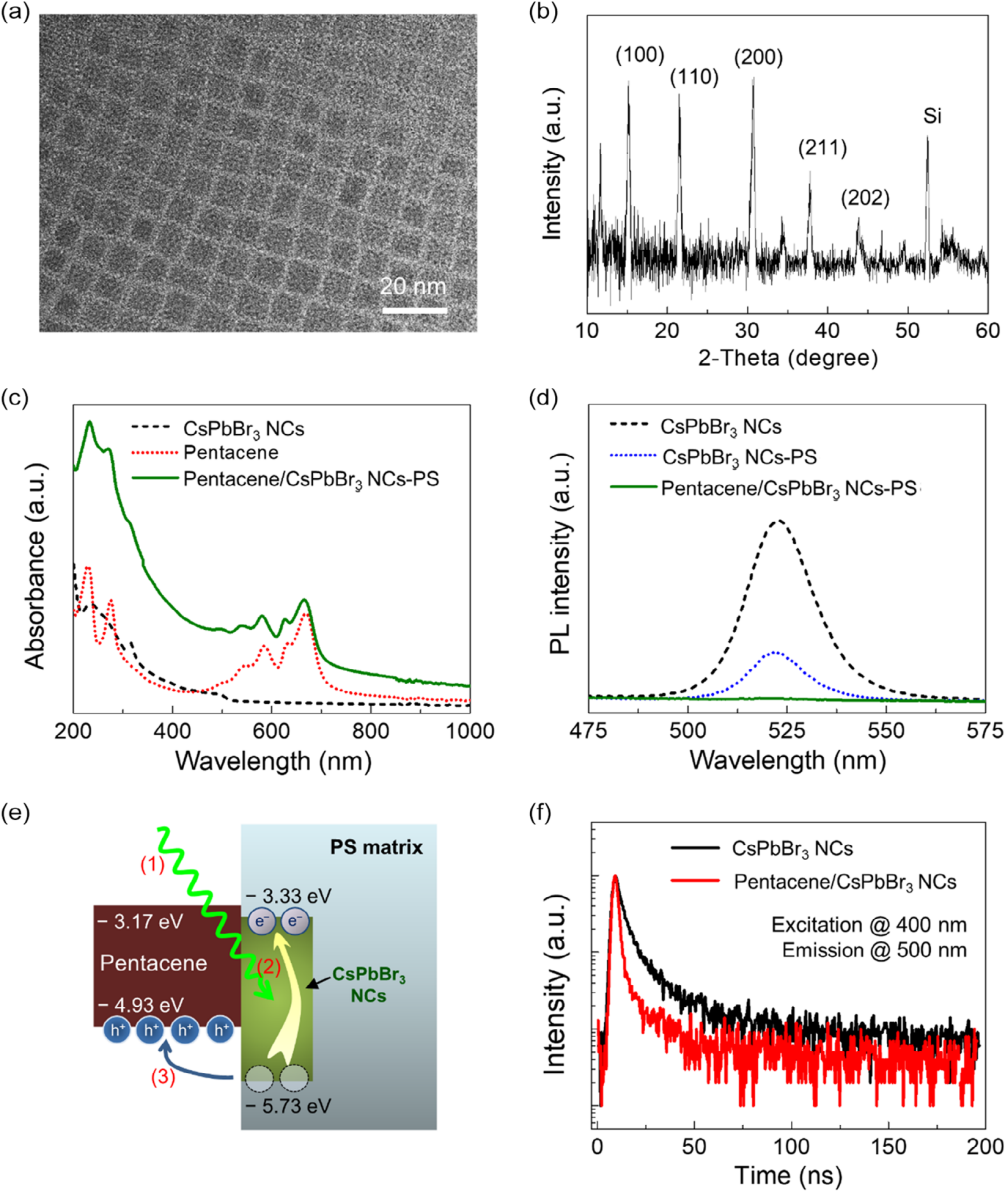
a) Transmission electron microscopy image and b) X‐ray diffraction patterns of the synthesized CsPbBr_3_ NCs. c) Absorbance and d) the photoluminescence intensities as a function of wavelength. e) Schematic of the charge transfer at the interface between pentacene and CsPbBr_3_ NCs in PS matrix due to the light absorption. (Process (1) light absorption, (2) electron–hole pairs photogeneration, (3) photoexcited hole charge transfer to pentacene). (f) Time‐resolved photoluminescence data of the synthesized CsPbBr_3_ NCs.

CsPbBr_3_ NCs were carefully synthesized *via* a conventional hot injection method for use in both the nano‐floating‐gate and the nanocomposite.^[^ ^42^ ^]^ After several purifications of the as‐synthesized CsPbBr_3_ NCs, we confirmed the successful synthesis of CsPbBr_3_ NCs through transmission electron microscopy images, as shown in **Figure** **2**a. The synthesized CsPbBr_3_ NCs had a cubic structure with an average size of 8.5 nm with a length deviation less than ±0.5 nm. Additionally, the crystalline structure of the CsPbBr_3_ NCs was further investigated by X‐ray diffractometer (XRD) analysis. From the XRD spectrum, Figure 2b, the positions of the peaks at 2*θ* = 15.13, 21.49, 30.67, 37.79, and 43.85 correspond to diffractions from (100), (110), (200), (211), and (202) plane peaks, which verified the cubic perovskite crystal structure of CsPbBr_3_ NCs.^[^ ^43^ ^]^ From the above results, we found that our CsPbBr_3_ NCs have excellent crystallinity with a cubic structure and uniformity in their size. Due to the structural properties of the CsPbBr_3_ NCs, we expected that they are reliable and predictable optoelectronic components which allow both charge trapping and light absorption in the ONFGTs. The optical properties of the CsPbBr_3_ NCs were investigated utilizing both UV–vis absorption and PL spectroscopies. Figure 2c presents the absorption spectra of the three different thin‐film structures with CsPbBr_3_ NCs (only), pentacene (only), and pentacene on CsPbBr_3_ NCs–PS nanocomposite on quartz substrates. We observed that the CsPbBr_3_ NCs had a band edge approximately located at 516 nm with a direct bandgap of about 2.40 eV, which was expected to be suitable for detection of the green and blue light. Pentacene, a p‐type organic semiconductor layer, exhibited an optical bandgap of 1.76 eV, which had a band edge located at 703 nm. Interestingly, the hybrid interface structure of pentacene/CsPbBr_3_ NCs–PS nanocomposite (green line in Figure 2c) showed an improved light absorption profile in all ranges below 700 nm because CsPbBr_3_ NCs and pentacene layers efficiently absorbed light of different wavelengths. The well‐dispersed CsPbBr_3_ NCs in the PS matrix at the structure of pentacene on the CsPbBr_3_ NCs–PS nanocomposite film seem to enhance the absorption spectrum profile in the range between 200 and 500 nm compared to that of the close‐packed CsPbBr_3_ NCs thin film. Figure 2d shows integrated PL intensities of the three different thin films with CsPbBr_3_ NC layers, a CsPbBr_3_ NCs–PS nanocomposite, and pentacene on a CsPbBr_3_ NCs–PS nanocomposite on quartz substrates over the range from 475 to 575 nm. The CsPbBr_3_ NCs exhibited photoemission spectra at 522 nm with a full‐width at half‐maximum (FWHM) of about 19.4 nm, which is due to the similar NC size distribution with a size deviation of ±0.5 nm as shown in the TEM image (Figure 2a). These optical properties of CsPbBr_3_ NCs well matched the measured energy levels by UPS, implying the coherence of the data. The PL peak of CsPbBr_3_ NCs–PS nanocomposite was observed in a similar peak position to the pristine CsPbBr_3_ NCs, indicating that the PS matrix did not affect the optical properties. The higher PL intensity of the CsPbBr_3_ NCs than that of CsPbBr_3_ NCs–PS composite originated from the higher concentration of the NCs. Note that the maximal PL intensity of the pentacene/CsPbBr_3_ NCs–PS nanocomposite structure was dramatically quenched to almost 9.7% compared to that of the CsPbBr_3_ NCs–PS nanocomposite layer. This was presumably due to the effective charge carrier transfer from the CsPbBr_3_ NCs–PS composite to the pentacene layer. Figure 2e shows the sequential photogenerated charge carrier transfer (Step (1)–(3)) at the interface between pentacene and CsPbBr_3_ NCs–PS nanocomposite. Since the CsPbBr_3_ NCs have a direct band structure, incident light with an energy larger than the bandgap of CsPbBr_3_ NCs generated photoexcited electron–hole pairs in the CsPbBr_3_ NCs–PS nanocomposite (Step (2) in Figure 2e). The recombined photoexcited carriers within the lifetime resulted in a PL peak corresponding to the bandgap of the CsPbBr_3_ NCs. In the pentacene/CsPbBr_3_ NCs–PS nanocomposite system shown in Figure 2e, the photoexcited holes in the CsPbBr_3_ NCs located at the interface were transferred to the adjacent pentacene because the energy potential of the HOMO of the CsPbBr_3_ NCs was higher than the HOMO of the pentacene (Step (3) in Figure 2e). The transferred holes flowed to the drain as the photocurrent by a hopping mechanism through the pentacene, which is the channel layer of the ONFGTs. Consequently, a relatively large amount of photoexcited electrons remained in the LUMO of the CsPbBr_3_ NCs.

To gain more insight into the limited electron–hole pair recombination and quenching processes in our system, we monitored the PL decay profiles of pristine CsPbBr_3_ NCs and pentacene/ CsPbBr_3_ NCs under excitation at 400 nm as shown in Figure 2f. The obtained results were fitted using a triexponential decay function: *I* = *A*_1_exp(−*t*/*τ*_1_) + *A*_2_exp(−*t*/*τ*_2_) + *A*_3_exp(−*t*/*τ*_3_)) and the average carrier lifetimes were calculated according to the equation: *τ*_av_ = (*A*_1_ *τ*_1_^2^ + *A*_2_ *τ*_2_^2^ + *A*_3_ *τ*_3_^2^)/(*A*_1_ *τ*_1_ + *A*_2_ *τ*_2_ + *A*_3_ *τ*_3_), where *τ*_1_, *τ*_2_, and *τ*_3_ are the fast, medium, and slow components of the decay time, respectively, and *A*_1_, *A*_2_ and *A*_3_ are their corresponding intensity parameters. Based on previous reports, both of the two faster decay components (*τ*_1_, *τ*_2_) can be assigned to trap‐mediated nonradiative recombination, whereas the slowest one (*τ*_3_) is primarily attributed to the intrinsic trap‐free charge recombination.^[^ ^44^ ^]^ As summarized in Table S1, Supporting Information, the charge carrier lifetime of the pentacene/CsPbBr_3_ NCs (9.8 ns) significantly decreased by about 72% compared with that of pristine CsPbBr_3_ NC films (35.3 ns), which are in good agreement with the PL quenching results. Considering these observations, we concluded that photogenerated excitons are well dissociated at pentacene/CsPbBr_3_ NCs interfaces, with the holes being rapidly and efficiently transferred to the pentacene layer.

**Figure 3:**
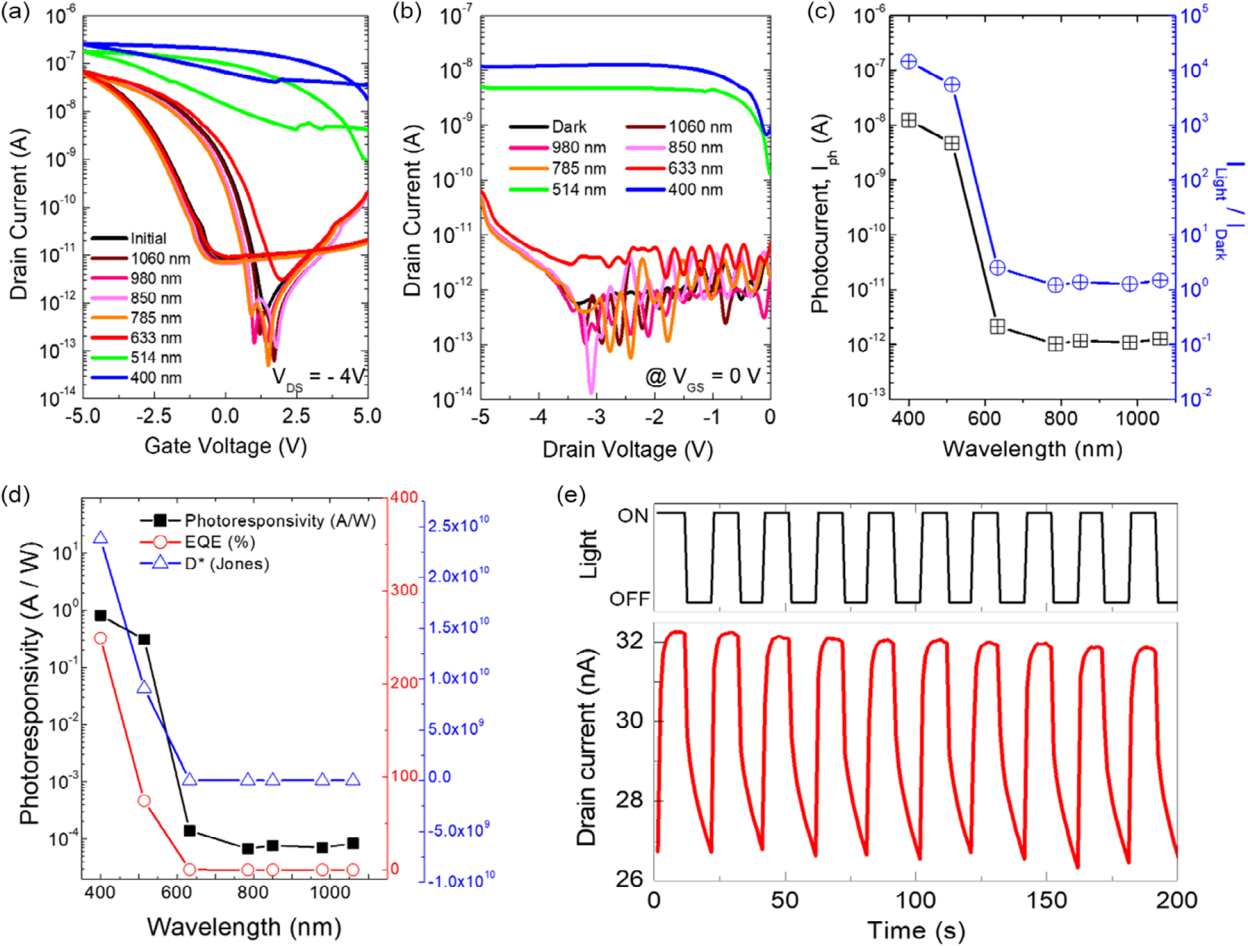
a) Transfer characteristics of the device with incident light wavelengths at *V*_DS_ = −4 V. b) Current–voltage curves under light irradiation with varying wavelengths at *V*_GS_ = 0 V. c) Photocurrent and *I*_Light_/*I*_Dark_ ratio as a function of light wavelength. d) Photoresponsivity (black line), EQE (red line), and specific detectivity (blue line) of the ONFGT as a function of the wavelength of incident light. e) Response of drain current of the ONFGTs against incident 514 nm of visible light with light intensity of 127 μW cm^−2^ at *V*_DS_ = −4 V, *V*_GS_ = 0 V.

To identify optoelectrical characteristics of the ONFGTs, we carried out transfer characteristic measurements (*I*_DS_–*V*_GS_) under irradiation of monochromatic light at different wavelengths over the range of 400–1080 nm using a tunable Xenon lamp light source with a holographic grating through an optical cable, as shown in **Figure** **3**. The transfer characteristics were measured at a *V*_DS_ of −4 V at a fixed incident illumination power of 76 μW cm^−2^ with various wavelengths under dark conditions. The black curve in Figure 3a indicates the initial transfer characteristics under dark conditions. Clockwise hysteresis was observed due to the trapping–detrapping of charge carriers in the CsPbBr_3_ NCs floating gate layer as a result of tunneling through the CsPbBr_3_ NCs–PS nanocomposite layer. It showed hysteresis windows of almost 2 V, indicating charge trapping memory transistor characteristics. It is noteworthy the validity of the CsPbBr_3_ NCs–PS nanocomposite layer in the ONFGTs. For comparison, we have tested the ONFGTs without a tunneling layer (Au/pentacene/CsPbBr_3_ NCs layer/blocking oxide/gate) and with only a PS tunneling layer (Au/pentacene/PS/CsPbBr_3_ NC layer/blocking oxide/gate) (see Figure S1, Supporting Information). As expected, the ONFGT without the CsPbBr_3_ NCs–PS nanocomposite tunneling layer exhibited significant hysteresis windows during the gate double sweep. Moreover, it demonstrated excellent photoresponse characteristics under light illumination, suggesting the efficient transfer of photogenerated charge carriers toward the semiconducting layer immediately after the absorption of photon energy. However, the device suffered from poor retention due to the absence of a tunneling layer, highlighting the necessity of an appropriate tunneling layer for nonvolatile memory functionality. We introduced a PS tunneling layer between the CsPbBr_3_ NCs layer (nano‐floating‐gate) and pentacene (semiconductor). The inclusion of the PS tunneling layer resulted in the manifestation of excellent hysteresis windows and improved retention characteristics. However, it is important to note that the photogenerated charge carriers at the CsPbBr_3_ NCs layer did not effectively transfer to the pentacene layer. This was evident from the minimal changes observed in the transfer curve of the transistor under both dark and light illumination conditions. In addition, we achieved reproducibly smooth surfaces of the CsPbBr_3_ NCs–PS nanocomposite layer with an RMS roughness of approximately 2.22 nm (see Figure S2, Supporting Information). Furthermore, we observed negligible differences in the device performance, including the photoresponse characteristics (see Figure S1, Supporting Information). It is widely recognized that the thickness of the tunneling layer exerts a significant influence on the memory characteristics. This is primarily due to the fact that a thinner tunneling layer facilitates easier transport of charge carriers from the pentacene (semiconductor) to the CsPbBr_3_ NCs layer (floating gate) under the same electrical field. Conversely, however, it also leads to a higher susceptibility of losing the stored charges, thereby implying poor retention characteristics. Moreover, it is crucial to take into account both the photoresponsivity and memory characteristics of the device, particularly when incorporating the CsPbBr_3_ NCs–PS nanocomposite layer between the pentacene and CsPbBr_3_ NC layer. By balancing these considerations, we have determined that an optimal tunneling layer thickness of approximately 23 nm is most suitable for our ONFGT. This choice ensures a favorable compromise between efficient charge transport and adequate memory characteristics.

We examined the photoresponsivity of the ONFGTs based on the above device characteristics in detail. The device was sequentially exposed to monochromatic light by changing its wavelength from 1080 to 400 nm. There were negligible changes in transfer characteristics of the ONFGTs for wavelengths of light down to 785 nm. This implies that the CsPbBr_3_ NCs at the nanocomposite layer do not respond to the incident light and generate sufficient charge carriers, which allows an increase in the conductance of the organic semiconducting layer of the ONFGTs. When the wavelength of light was 633 nm, we observed slight changes in transfer characteristics as shown in Figure 3a. This partially contributed to the drain current of the ONFGTs due to the generation of photocurrent by absorbing the light at the pentacene layer (1.76 eV, λ = 704 nm). On the other hand, the ONFGTs exhibited a significant increase of drain current below 514 nm light irradiation, which resulted from the considerable photocurrent that originated from the strong absorption of the light at CsPbBr_3_ NCs in the CsPbBr_3_ NCs–PS nanocomposite layer. These results suggested that the CsPbBr_3_ NCs absorb light more efficiently than pentacene and the photoinduced charge carriers in CsPbBr_3_ NCs transferred to the pentacene active layer and flowed as a photocurrent. The photocurrent below 514 nm boosted both the ON and the OFF current with larger hysteresis windows than that under dark conditions. These results indicated that the photoinduced electrons could be effectively trapped in the CsPbBr_3_ NCs located between Al_2_O_3_ and the nanocomposite layer with the application of a gate voltage, and the trapped charge could not be released without the application of a strong negative gate voltage. The trapped photoelectrons that were not recombined in the CsPbBr_3_ NCs show an effective negative gating behavior for the pentacene channel, thereby increasing the hole currents through capacitive coupling in the pentacene. The photoelectron trapping in CsPbBr_3_ NCs under light irradiation can be understood as similar to the charge trapping by a positive gate bias. Additionally, we examined the output characteristics of the ONFGTs at *V*_GS_ = 0 V under light irradiation with the decreasing wavelength from 1080 to 400 nm, as shown in Figure 3b. As expected and shown in the transfer characteristics above, negligible photocurrent was observed under relatively long wavelength light (longer than 633 nm). On the other hand, the significant drain current flow was monitored up to ≈10^−8^ A at 400 nm. For further analysis, we calculated photocurrent (*I*_ph_ = *I*_light_ – *I*_dark_, black line) and the current ratio of the photocurrents to the dark current (blue line) as a function of wavelengths of irradiated light at *V*_DS_ = −3 V and *V*_GS_ = 0 V, as shown in Figure 3c. Most of the current flow from the source to drain electrode was due to the photoinduced charge carriers that originated from the CsPbBr_3_ NCs in the interfacial CsPbBr_3_ NC‐PS nanocomposite layer. Our ONFGTs exhibited significant changes in both the photocurrent (*I*_ph_) and the *I*_lignt_/*I*_Dark_ ratio of 10^−8^ A and 10^4^, respectively, suggesting excellent selectivity of visible light under a wavelength of 600 nm. These electrical properties of the ONFGTs are expected to enable a photomemory capable of storing optical signals as they show completely different characteristics compared to the typical photodetectors whose instantaneous current changes with incident light. Although all measurements were carried out under ambient conditions, we have not observed any significant degradation in performance. However, in order to enhance the stability of the ONFGT, it is advisable to store it in an inert environment or utilize robust encapsulation techniques. These will help safeguard the device against potential degradation and ensure its long‐term stability.

Furthermore, to quantitatively measure the photoelectric performance of our ONFGTs, their photoresponsivity (*R*) and external quantum efficiency (EQE) were calculated as a function of incident light wavelength using the following equations^[^ ^36^, ^45^ ^]^

(1) $$R=\;\frac{I_{ph}}{PWL}$$

(2) $$\text{EQE}=\frac{Rhc}{\lambda e}$$

where *P* is the light power incident on the surface of the device, *W* and *L* are the width and length of the irradiated area, respectively, *h* is the Plank's constant, *λ* is the incident light wavelength, *c* is the speed of light in a vacuum, and *e* is the electronic charge.

According to the earlier equations, the *R* and EQE of our device were calculated and then displayed in Figure 3d, where the maximum *R* and EQE were 0.804 AW^−1^ and 249.3% under illumination at 400 nm, respectively. Similar to the *I*_ph_ trend, our device reveals a strong/selective response in the blue‐green region of the visible spectrum that is consistent with the absorption profile of the CsPbBr_3_ NCs, suggesting NCs provide the main contribution to photocurrent generation in our ONFGT. Also, we have summarized the performance parameters of nanosized CsPbBr_3_‐based photodetectors reported in prior studies (Table S2, Supporting Information). It is noteworthy that the photoelectric performance of our device is found to be comparable to or even higher than that of previously reported devices in the literature, which can be attributed to the efficient extraction and transport of photogenerated charge carriers at the interface between CsPbBr_3_ NCs and pentacene layer.

Another important parameter used to evaluate photodetector performance is the specific detectivity (D*), which describes the capability of detecting the weakest light signal and is defined by the following equation^[^ ^36^ ^]^

(3) $$D^{*}=\frac{R\sqrt{A\Delta f}}{I_{n}}$$

where *A* is the effective area of the photodetector, *Δf* is the electrical bandwidth, and *I*_*n*_ is the root‐mean‐square value of the measured noise current.

As shown in Figure 3d, this photoresponse parameter exhibits almost the same evolution trend as both *R* and EQE, with the highest value of 2.38 × 10^10^ jones with excitation at 400 nm, which are comparable to the values reported in the literature for perovskite‐based photodetectors.^[^ ^46^, ^47^, ^48^ ^]^ Given the results obtained from PL and TRPL measurements, such a high D* was ascribed to a decrease in dark current due to efficient photogenerated charge carrier extraction and transport at the pentacene/CsPbBr_3_ NCs. Based on the above photoresponsivity of our ONFGTs, we examined the repeated photoresponsivity against incident 514 nm UV light with an intensity of 127 μW cm^−2^, as shown in Figure 3e. According to the turn on and off the visible light, the channel resistance states of the ONFGTs are modulated by generation and transport of the charge carriers from the perovskite NCs to the semiconductor layer. The drain current of the ONFGTs was measured to be 26.3 and 32.2 nA for turn off and on of the light, respectively. The repeated and reversible photoresponsivity of the device implies that our ONFGTs are applicable for photodetection devices.

**Figure 4:**
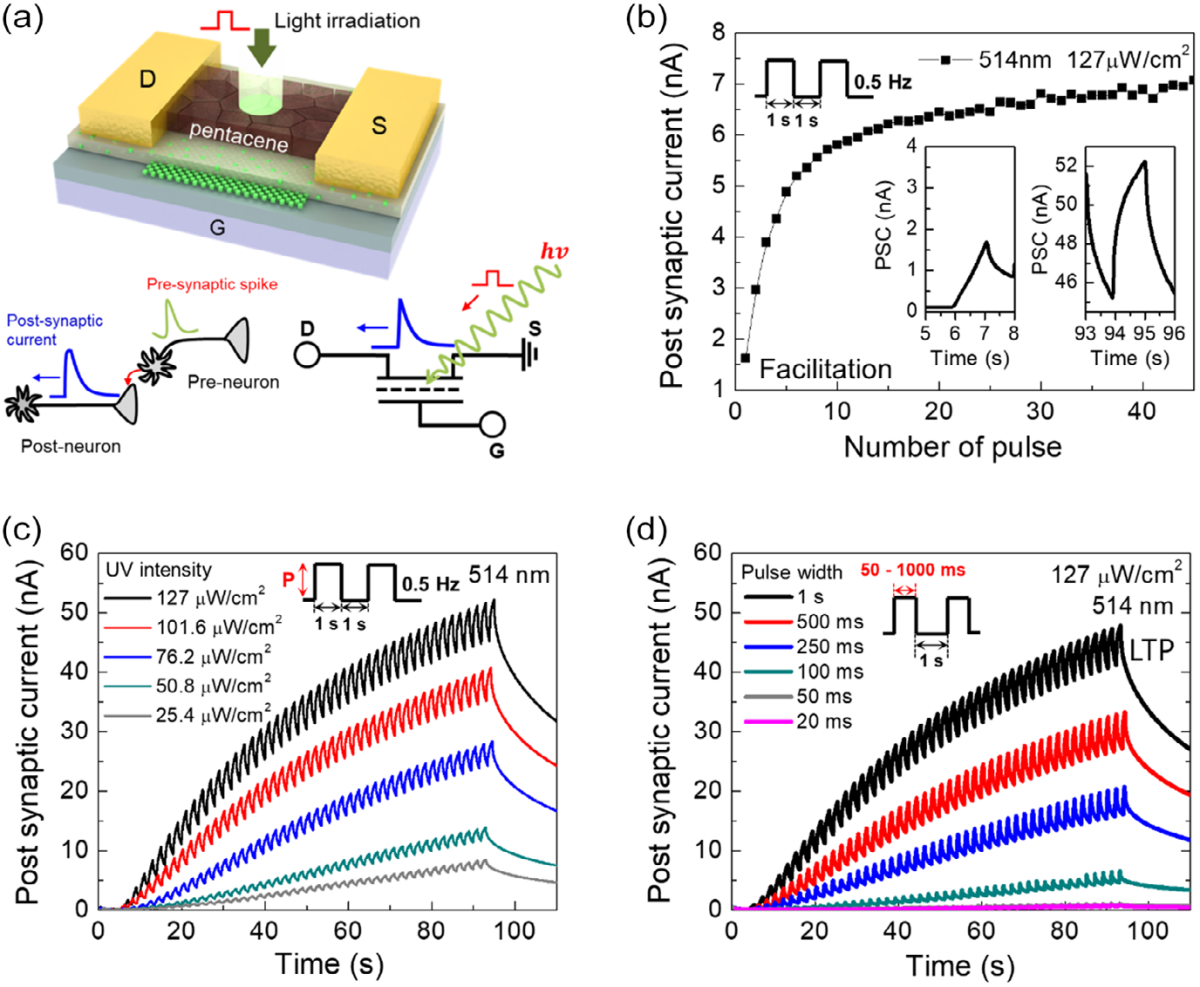
a) Schematics of the ONFGT under light illumination, signal transmission in a biological synapse (left) and the corresponding equivalent circuit of the ONFGT synapse. b) Change of synaptic weight per visible‐light pulse emulating the neural facilitation. c) PSC of the ONFGT synapse as a function of the intensity of visible‐light pulse (from 25.4 to 127 μW cm^−2^). d) PSC of the ONFGT synapse as a function of the width of visible‐light pulse (from 20 to 1000 ms).

Based on the above photoresponsivity of the ONFGTs, we examined photosynaptic characteristics against incident visible light as shown in **Figure** **4**a. Because the ONFGTs exhibited optimum photocurrent under 514 nm of light excitation, dynamic modulation of the photocurrent of the ONFGTs was implemented by varying the intensity and duration time of the light, which is an important factor for the demonstration of photonic synaptic applications. In biological nervous systems, neurons communicate with each other through electrical and chemical signals. When a neuron receives enough stimulation, it generates an electrical signal called an action potential, which travels down its axon and releases neurotransmitters at its synapses, which then activate or inhibit other neurons.^[^ ^49^ ^]^ The operation of biological synapses begins with action potentials traveling to the axon of the presynaptic neuron. Then, when the action potential reaches the end of the axon, it triggers the release of a chemical called a neurotransmitter into the synaptic cleft, which is the small gap between the presynaptic neuron and the postsynaptic neuron. Finally, the neurotransmitter diffuses across the synaptic cleft and binds to receptors on the postsynaptic neuron. In this study, the light signal was introduced as an external stimulus to modify the synaptic weight which is called synaptic plasticity. It is well known that the amplitude of the PSC of the post‐neuron is obviously proportional to the synaptic weight.

To investigate light‐driven synaptic plasticity of the ONFGTs at constant *V*_DS_ of −4 V, the PSC was monitored by an excitation wavelength of 514 nm with an intensity of 127 μW cm^−2^, as shown in Figure 4b. The synaptic strength is generally determined by the amplitude of the PSC in the post‐neuron (post‐synaptic) during propagation of single input spike in the preneuron (presynaptic). The post‐neuron was excited by the sufficient input spike (visible‐light pulse) to the pre‐neuron. The PSC of the device responded by increasing 1.7 nA against the initial single visible‐light pulse as shown in the inset of Figure 4b. After the 45th pulse signal train with 0.5 Hz, the PSC value (synaptic weight) increased by 7.0 nA, which is almost four times higher than that of the first response. By plotting the changes in the synaptic weight per each light pulse, we clearly observed an increase in synaptic weight under repeated photonic stimulations. This implies that CsPbBr_3_ NCs generate charge carriers under irradiation of visible pulse and decrease channel resistance, resulting in enhanced PSC values. Additionally, we examined the PSCs under different intensities of light illumination with 514 nm as shown in Figure 4c. By varying the intensity of visible light from 25.4 to 127 μW cm^−2^, the PSCs changed to become proportional to the light intensity under each irradiation condition. Because the synaptic strength is generally determined by the amplitude of the PSC, the pulse train of visible light with 127 μW cm^−2^ enhanced the stimulation of the pre‐neuron, resulting in higher PSC of 52 nA after the 45th light pulse irradiation. Similarly, the PSC was controllable depending on the pulse width (duration time) of the visible‐light pulse, as shown in Figure 4d. As expected, the change of the PSC amplitude gradually increased under the sufficient stimulation of the pre‐neuron with longer firing time than shorter case. It also exhibited long‐term potentiation (LTP) characteristics by mimicking the excitatory synapse.

**Figure 5:**
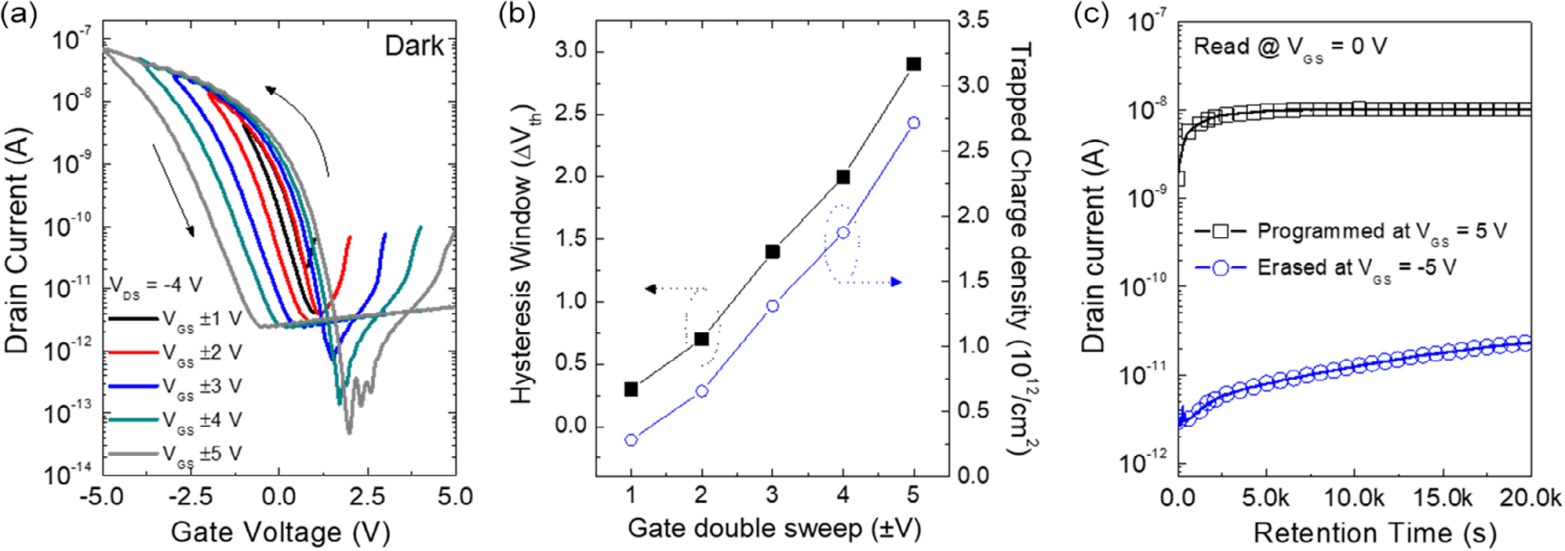
a) Transfer characteristics of the ONFGT with CsPbBr_3_ NCs at *V*_DS_ = −4 V. b) Hysteresis windows and the total trapped charge densities as a function of applied gate voltage range. c) Retention time characteristics of the ONFGT. All electrical measurements were carried out in the dark.

Additionally, we investigated nonvolatile memory characteristics of the ONFGTs. **Figure** **5**a shows the apparent counterclockwise hysteresis behavior of the transfer characteristics of the ONFGTs. These hysteresis characteristics are typical of charge trap memories due to the charge carrier trapping in the CsPbBr_3_ NCs layer between the CsPbBr_3_ NCs–PS nanocomposite layer and Al_2_O_3_ as the gate bias is applied. The asymmetric hysteresis of the transfer curves (Figure 5a) occurred because the trapping of holes, which were majority carriers in pentacene, were easier than the trap of electrons in the CsPbBr_3_ NCs floating gate. Figure 5b displays the hysteresis windows and the total trapped charge densities as a function of applied gate voltage range. The ONFGTs did not show any meaningful hysteresis window at an initial gate bias (*V*_G_) double sweep of ±1.0 V, implying negligible charge carrier trapping in the CsPbBr_3_ NCs layer. By increasing the gate bias double sweep from ±1.0 to ±5.0 V with ±1 V step, the corresponding hysteresis window value (Δ*V*_th_) was gradually increased from 0.3 to 2.9 V as shown in the black square box of Figure 5b. To evaluated how much carriers can be stored in the CsPbBr_3_ NCs layer, we calculated the trapped charge density in the CsPbBr_3_ NCs floating gate using the following equation

(4) $$n=\frac{\left|\Delta V_{\text{th}}\right|\times C_{i}}{e}$$

where *n* is the trapped charge density per unit area of the CsPbBr_3_ between the nanocomposite tunneling layer and Al_2_O_3_ dielectric layer, Δ*V*_th_ is the hysteresis window of the device, *C*_*i*_ is the capacitance per unit area of the dielectric layer, and *e* is the elementary electric charge (1.602 × 10^−19^ C). The measured capacitance per unit area of the dielectric (CsPbBr_3_ NCs–PS nanocomposite/CsPbBr_3_ NCs layer/Al_2_O_3_) was 150 nF cm^−2^. After the programming and erasing event, we found that the calculated trapped charge density was almost 2.81 × 10^11^ cm^−2^ at *V*_G_ = ±1 V. As gate double sweep voltage increased, the charge density was gradually increased and achieved 2.71 × 10^12^ cm^−2^ at *V*_G_ = ±5 V. The retention time characteristics were measured, as shown in Figure 5c. After writing (*V*_G_ = +5 V) and erasing (*V*_G_ = −5 V), we monitored the drain current continuously at *V*_G_ = 0 V and *V*_DS_ = −5 V with intervals of 4 s. Their drain current values were well maintained after programing and erasing, exhibiting values of more than two orders of magnitude for 20 000 s. Due to the considerable retention characteristics of our ONFGTs, the charge carriers are expected to be trapped mostly the CsPbBr_3_ NCs floating gate layer rather than at the CsPbBr_3_ NCs–PS nanocomposite tunneling layer. These results indicate that the CsPbBr_3_ NCs layer provides charge storage trapping sites and can be considered as a potential storage medium to implement low voltage nonvolatile memory devices.

## 3 Conclusions

We have implemented an independent dual‐function ONFGT with photosynaptic and electrical memory functions by introducing a perovskite (CsPbBr_3_) NCs–insulating polymer (PS) nanocomposite as a tunneling layer and a CsPbBr_3_ NC layer as a floating gate layer. Our perovskite NC‐based nano‐floating‐gate transistor responded under irradiation, implying that photogenerated charge carriers transferred from CsPbBr_3_ NCs of the nanocomposite to the organic semiconductor layer. Based on the photoresponsivity of the device, we examined photosynaptic characteristics that mimic the biological nervous system. The dynamic modulation of the photocurrent of the device was implemented by varying the intensity and duration of the light. By plotting the changes in the synaptic weight per each visible‐light pulse, we clearly observed an increase in synaptic weight (PSC) under repeated photonic stimulation. Additionally, the perovskite NC‐based ONFGT exhibited excellent nonvolatile memory characteristics displaying a 2.9 V hysteresis window value (Δ*V*_th_) for a gate bias double sweep under ±5.0 V. Drain current values were well maintained after programing and erasing, exhibiting values of more than two orders of magnitude for 20 000 s. Finally, we concluded that the perovskite NCs–insulating polymer nanocomposite tunneling layer is a key component for achieving dual functions (photoresponsivity and memory characteristics) on conventional nano‐floating‐gate transistors.

## 4 Experimental Section

Cesium carbonate (Cs_2_CO_3_, 99.9%), oleic acid (OA, 90%), oleylamine (OLA, 70%), and PS (average Mw ≈192 000) were purchased from Sigma‐Aldrich. Lead(II) bromide (PbBr_2_, 99.999%) and octadecene (ODE, 90%) were purchased from Thermo‐Fisher. All chemicals were used without further purification. Cs_2_CO_3_ (250 mg) and 1 mL of OA along with 10 mL of ODE were added into a 100 mL 3‐neck flask in an oil bath. The mixture was purged for 2 h at 120 °C to remove moisture. The solution was heated to 150 °C and maintained for 30 min under an N_2_ atmosphere to dissolve Cs_2_CO_3_ completely and react with OA. It was maintained above 105 °C to prevent precipitation of Cs‐oleate before injection. Then, 20 mL of ODE and 276 mg of PbBr_2_ were loaded into a 100 mL 3‐neck flask and were purged with N_2_ at 120 °C for 1 h. Next, 2 mL of OA and 2 mL of OLA were injected at 120 °C under N_2_ purge. After complete dissolution of PbBr_2_, the solution was heated to 150 °C and 1.6 mL of the prepared Cs‐oleate solution was quickly injected into the PbBr_2_ solution. After the synthesis reaction of CsPbBr_3_ quantum dots (NCs) for 30 s, the mixture was cooled down in an ice bath. The synthesized CsPbBr_3_ NCs solution was cooled down with a water bath and remaining reactants and by‐products were separated by centrifuging at 15 000 rpm at 20 °C for 30 min. After removing the supernatant, the precipitates were dispersed in toluene and then centrifuged again at 8000 rpm at 4 °C for 20 min. Finally, the bright green CsPbBr_3_ solution in toluene was obtained by filtration of a supernatant using a 200 nm membrane filter to discard the aggregated NCs.

A heavily n‐doped bare Si (100) wafer was diced, cleaned using ultra‐sonication in acetone and isopropanol for 10 min, and then dried in convection oven at 70 °C for 30 min. Al_2_O_3_ dielectric films were deposited on the prepared substrates by 200 ALD cycles (Lucida D100 ALD, NCD) at 200 °C. Trimethylaluminum (Al(CH_3_)_3_) and water were used as the Al and O precursors, respectively. The growth rate per cycle was calculated to be ≈0.12 nm cycle^−1^. The CsPbBr_3_ NCs solution was spin‐coated on the Al_2_O_3_ deposited substrates at 1000 rpm for 1 min and were then baked at 180 °C for 30 s in glove box. The spin‐coating and baking procedures were repeated four times in total. To prepare the CsPbBr_3_ NCs/PS composite film, 3 mg of PS was added to the 1 mL of the CsPbBr_3_ NCs solution, and it was stirred for 2 h. The CsPbBr_3_ NCs/PS composite solution was spin‐coated at 4000 rpm and was baked at 180 °C for 30 s in a glove box. Pentacene films (50 nm thick) were evaporated onto the CsPbBr_3_ NCs/PS composite layer using a shadow mask with a deposition rate of 0.2–0.3 Å s^−1^ at 10^−7^ Torr. Finally, gold source/drain electrodes (50 nm thick, channel width *W* = 1000 μm and channel length *L* = 15 μm) were thermally evaporated at a pressure of 10^−6^ Torr.

Electrical characterization was performed using a semiconductor parameter analyzer (Keithley 4200SCS) under ambient condition. The chemical features of the films were identified by X‐ray photoelectron spectroscopy (XPS, K‐Alpha, Thermo Fisher Scientific). The successful synthesis of CsPbBr_3_ NCs was confirmed using a transmission electron microscope (TEM, Tecnai G2‐F20 460 L, FEI) with an acceleration voltage of 200 kV. The crystalline structures of the CsPbBr_3_ NCs were investigated with Cu Kα irradiation for 2*θ* values ranging from 10° to 60° using a laboratory XRD (Empyrean, Malvern Panalytical). UV–Vis absorption spectra for thin films were collected using a Jasco V670 spectrometer in transmission mode. The PL spectra were obtained using the Horiba NanoLog‐C spectrofluorometer system. Time‐resolved photoluminescence was performed to analyze the photocarrier lifetime of the CsPbBr_3_ NCs/pentacene hybrid system. The second harmonic (SHG = 400 nm) of a tunable Ti:sapphire laser (MaiTai, Spectra Physics) with an ≈100 fs pulse width and 80 MHz repetition rate was used as the excitation source. XPS (Thermo Scientific ESCALAB 250Xi) using monochromated Al Kα (1486.6 eV) was employed for the produced CsPbBr_3_ NCs containing relative amounts of nitrogen groups. The spot size of the X‐ray was 200 μm, and the instrument resolution was ≈0.1 eV with a pass energy of 50 eV.

## Conflict of Interest

The authors declare no conflict of interest.

## Supporting information

Supplementary Material

## Data Availability

The data that support the findings of this study are available from the corresponding author upon reasonable request.
