# Supplementary material for: Implementation of Photosynaptic and Electrical Memory Functions in Organic Nano‐Floating‐Gate Transistors via a Perovskite‐Nanocrystal‐Based Nanocomposite Tunneling Layer
Source: Small Sci. 2023 Jul 18;3(9):2300068. doi: 10.1002/smsc.202300068 (PMC11935999; doi:10.1002/smsc.202300068)
Supplement: Supplementary file 1 — Supplementary Material [file SMSC-3-2300068-s001.pdf]

Supporting Information

Implementation of Photosynaptic and Electrical Memory Functions in Organic Nano-Floating-Gate Transistors via a Perovskite-Nanocrystal-Based Nanocomposite Tunneling Layer

Byung Joon Moon, Young Seok Song, Dabin Son, Sukang Bae, Seoung-Ki Lee, Sang Hyun Lee and Tae-Wook Kim**

Figure S1. Schematics, transfer characteristics, photo-response and retention of the different structures of ONFGTs.

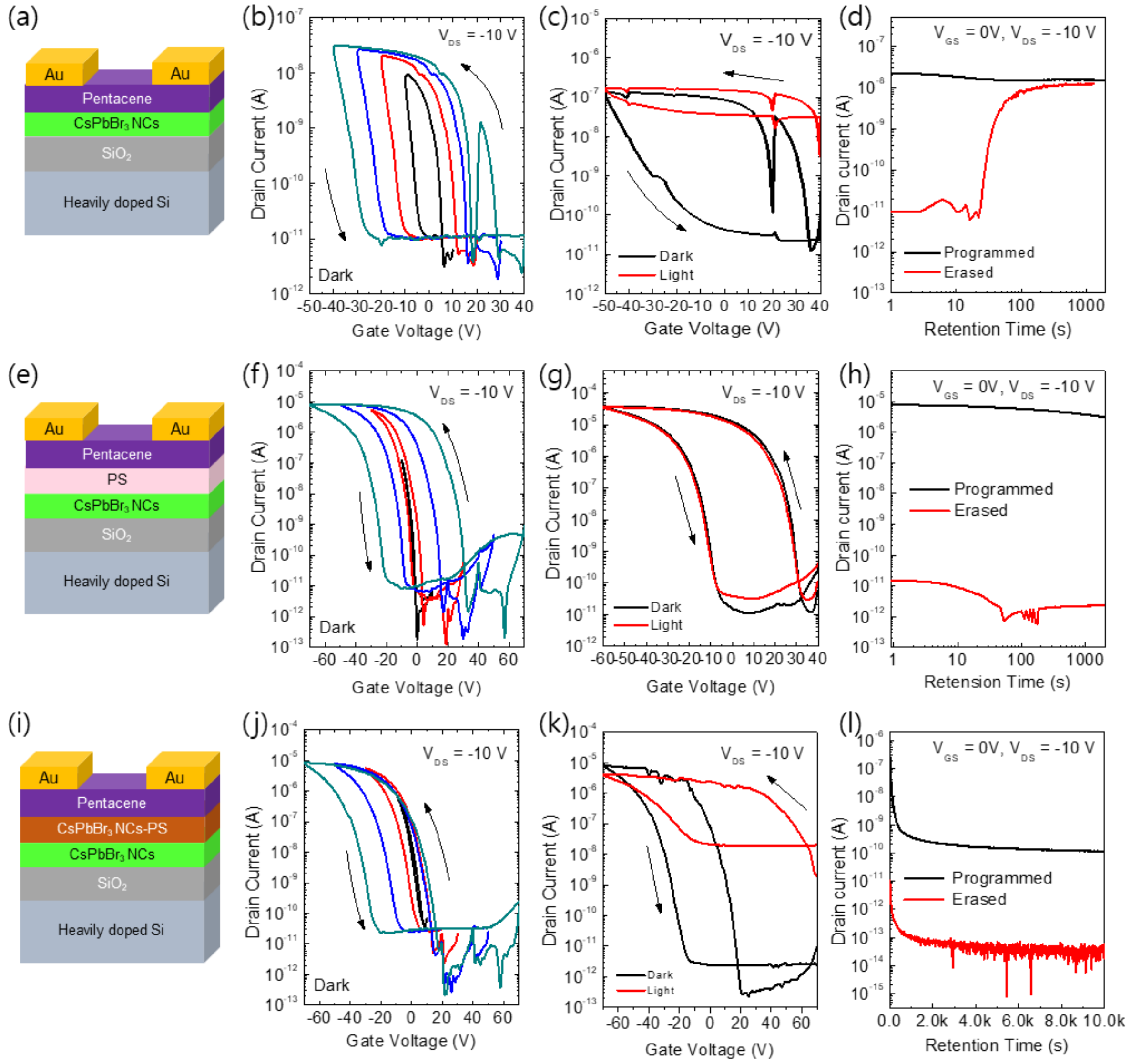

Figure S2. AFM image of (a) $CsPbBr_3$ NCs layer on SiO_2/Si and (c) $CsPbBr_3$ NCs-PS nanocomposite layer on $CsPbBr_3$ NCs layer/SiO_2/Si. Transfer characteristics of ONFGT (b) without and (d) with $CsPbBr_3$ NCs-PS nanocomposite layer.

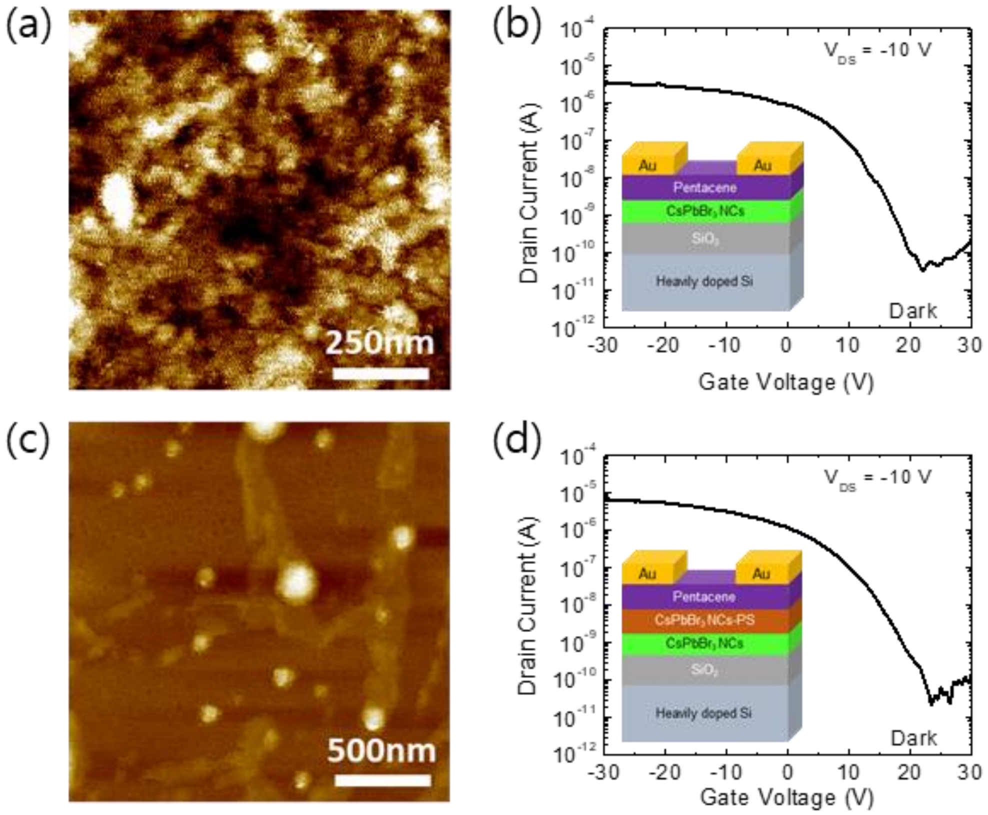

Table S1. PL lifetimes of $CsPbBr_3$ NCs and $CsPbBr_3$ NCs/pentacene films (substrate: Si/SiO_2)

	A_1	τ_1 (ns)	A_2	τ_2 (ns)	A_3	τ_3 (ns)	τ_{avg} (ns)
**$CsPbBr_3$**	0.04	3.226	0.003	11.530	0.003	121.976	35.27
**$CsPbBr_3$/Pentacene**	0.091	2.087	0.001	4.479	0.001	78.652	9.79

Table S2. Performance parameters of $CsPbBr_3$-based photodetectors

	Devices Structure	Parameters			Ref
		Responsivity ($A\ W^{-1}$)	EQE (%)	I_{illu}/I_{dark}	
only $CsPbBr_3$	Au/$CsPbBr_3$ NCs/Si	0.01	8	10^5	[1]
	Pt/$CsPbBr_3$ NCs/Au	0.028	6	10^5	[2]
	$CsPbBr_3$ NCs/ITO	0.64	54	10^3	[3]
	Au/$CsPbBr_3$ NSs:Au NPs/Si	0.097	22.2	8.5×10^3	[4]
$CsPbBr_3$ /charge transport layer	Au/MoO_3/$CsPbBr_3$ NCs/ZnO/FTO	0.3	-	4.5×10^2	[5]
	Au/PbS QDs/$CsPbBr_3$ QDs/PEODT:PSS/ITO	0.001	-	10^3	[6]
	Ag/P3HT/PDPP3T/$CsPbBr_3$ NWs/SnO_2/ITO	0.25	-	-	[7]
$CsPbBr_3$ /dielectric layer	Au/$CsPbBr_3$ NSs/SiO_2/Si	0.53	-	-	[8]
	Au/$CsPbBr_3$ NCs/SiO_2/Si	0.18	41	8×10^3	[9]
	Au/Pentacene/$CsPbBr_3$ NCs-PS/$CsPbBr_3$ NCs/Al_2O_3/Si	0.804	249.3	10^4	This work

Reference

1. Y. Dong *et al.*, *Small* **2016**, *12*, 5622.
2. M. I. Saidaminov *et al.*, *Adv. Optical Mater.* **2017**, *5*, 1600704.
3. J. Song *et al.*, *Adv. Mater.* **2016**, *28*, 4861.
4. Y. Dong *et al.*, *Adv. Mater. Interfaces* **2021**, *8*, 2002053.
5. M. Xue *et al.*, *Solar Energy Materials and Solar Cells* **2018**, *187,* 69.
6. H. Zhao *et al.*, *Nanotechnology* **2020**, *31*, 035202.
7. F. Cao *et al.*, *Adv. Funct. Mater.* **2019**, *28*, 1906756.
8. Z. Yang *et al.*, *Adv. Funct. Mater.* **2018**, *28*, 1705908.
9. X. Li *et al.*, *Adv. Funct. Mater.* **2016**, *26*, 5903.
